# In Vivo Models for Prostate Cancer Research

**DOI:** 10.3390/cancers14215321

**Published:** 2022-10-28

**Authors:** Robert Adamiecki, Anita Hryniewicz-Jankowska, Maria A. Ortiz, Xiang Li, Baylee A. Porter-Hansen, Imad Nsouli, Gennady Bratslavsky, Leszek Kotula

**Affiliations:** 1 Rutgers New Jersey Medical School, Rutgers University, Newark, NJ 07103, USA; 2 Department of Urology, SUNY Upstate Medical University, 750 East Adams Str., Syracuse, NY 13010, USA; 3 Department of Cytobiochemistry, Faculty of Biotechnology, University of Wroclaw, ul. F. Joliot-Curie 14a, 50-383 Wroclaw, Poland; 4 Department of Biochemistry and Molecular Biology, SUNY Upstate Medical University, 750 East Adams Str., Syracuse, NY 13010, USA; 5 Upstate Cancer Center, SUNY Upstate Medical University, 750 East Adams Str., Syracuse, NY 13010, USA

**Keywords:** prostate cancer, knockout mouse models, genetically-engineered mouse models, xenografts, patient derived xenografts, organoids, signaling pathways

## Abstract

**Simple Summary:**

This review explores in vivo models of prostate cancer currently published in the literature, with the focus on the prostate cancer mouse models that have recently been proposed. The information that researchers currently have about such models is critical for the information that they hope to obtain from future studies. Therefore, it is important that the various models currently published in the literature are systematically brought together. With the benefits and drawbacks of various types of prostate cancer models provided in this review, combined with their relationships to different signaling pathways and stages of tumor progression, the researcher may tackle the question of which model or gene of interest associated with the development of prostate cancer bests suits their future studies.

**Abstract:**

In 2022, prostate cancer (PCa) is estimated to be the most commonly diagnosed cancer in men in the United States—almost 270,000 American men are estimated to be diagnosed with PCa in 2022. This review compares and contrasts in vivo models of PCa with regards to the altered genes, signaling pathways, and stages of tumor progression associated with each model. The main type of model included in this review are genetically engineered mouse models, which include conditional and constitutive knockout model. 2D cell lines, 3D organoids and spheroids, xenografts and allografts, and patient derived models are also included. The major applications, advantages and disadvantages, and ease of use and cost are unique to each type of model, but they all make it easier to translate the tumor progression that is seen in the mouse prostate to the human prostate. Although both human and mouse prostates are androgen-dependent, the fact that the native, genetically unaltered prostate in mice cannot give rise to carcinoma is an especially critical component of PCa models. Thanks to the similarities between the mouse and human genome, our knowledge of PCa has been expanded, and will continue to do so, through models of PCa.

## 1. Introduction

### Historical Timeline of PCa Models

In the United States, prostate cancer is estimated to be the most commonly diagnosed cancer in men in 2022 [1]. The level of genetic information we have obtained over past decades of research has guided the development of models that attempt to study PCa. When cancer cell lines were first developed in the late 1940s through the 1970s, information about their genetic makeup or mutations present in the malignant tumor cells was limited. Cancer cells were simply taken from either the primary tumor or metastasis and were grown in vitro, allowing researchers to obtain ‘immortal’ cells by passaging them over time. The first cell line, or the L929 cell line, was established by Dr. Wilton R. Earle in 1948 and was derived from fibroblasts in subcutaneous mouse tissue [2]. The first immortalized human cell line, the HeLa cell line developed by Dr. George O. Gay in 1951, used cells taken from epithelial cervix tissue from Henrietta Lacks [3]. Many more cell lines soon followed throughout the 1950s and 1960s that were taken from hamsters, canines, monkeys, and humans [4]. Important PCa cell lines—LNCaP cells that were taken from a lymph node metastasis [5], DU145 cells that were taken from a central nervous system metastasis [6], and PC3 cells that were taken from a bone metastasis [7]—were developed in 1977, 1978, and 1979, respectively. Despite the cancer researcher’s ability to finally grow malignant, immortalized cells in vitro, little was known about the cells’ gene expression or the mutations that promoted their tumorigenicity.

**Figure 1:**
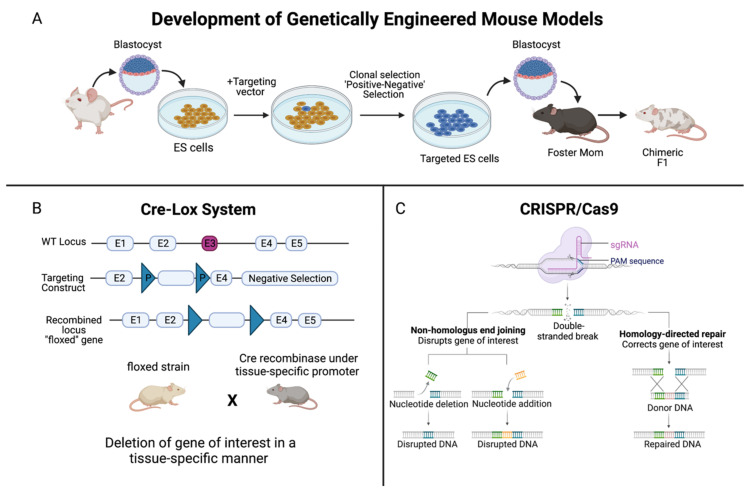
Summary of the development of genetically-engineered mouse models. (**A**) Genetically-engineered (GE) mice are used extensively in research as models of human disease. In the context of cancer, by deleting, or “knocking out”, the function of a specific gene, one can determine the significance of that gene in either promoting or halting the progression of tumor development. The method to develop GE models begins with extracting blastocysts from the mouse, and culturing embryonic stem (ES) cells. A targeting vector, which is a DNA construct containing DNA sequences homologous to the gene of interest, is added. ES cells that successfully recombine with the genomic DNA are selected for, and these targeted ES cells are injected into the mouse blastocysts. (**B**) The Cre-Lox system is used to control site specific recombination events in genomic DNA, allowing one to control expression of a specific gene or to delete undesired sequences. Cre recombinase is an enzyme that is derived from the bacteriophage P1, and it catalyzes a site-specific recombination event between two DNA recognition sites known as LoxP sites [9]. The result is a recombined locus in which the gene of interest is deleted, creating a “floxed” gene. (**C**) In the CRISPR/Cas9 system, a foreign single guide RNA (sgRNA) seeks, matches, and binds a specific DNA sequence, and the nuclease Cas9 cuts the sequence at a precise binding site near a protospacer adjacent motif (PAM) sequence [10]. The double-stranded DNA break induced by Cas9 is repaired by the cell’s DNA repair machinery. One method is via non-homologous end joining, either by deleting or adding a nucleotide, which does not require a template but disrupts the gene of interest. Or, homology-directed repair can be performed, which corrects the gene of interest, but a single stranded (ss) DNA template is required, which is provided by the CRISPR/Cas9 system [10]. Created with BioRender.com.

PCa models, as well as our understanding of several aspects of mammalian physiology, were significantly enhanced thanks to the research by Mario R. Capecchi, Sir Martin J. Evans, and Oliver Smithies in the late 1980s. In 2007, these scientists were jointly awarded The Nobel Prize in Physiology or Medicine for discovering how to introduce specific gene modifications in mice using embryonic stem cells [8]. Through homologous recombination between introduced DNA and endogenous DNA in embryonic stem cells, pure populations of cells carrying the target gene could be grown and injected into blastocysts [8]. The injected blastocysts are implanted into a surrogate mother where they develop into mosaic embryos, and then mosaic and normal mice mate to produce both gene targeted and normal offspring [8]. This powerful technique, known as gene targeting in mice, is often used to inactivate, or knock out, specific genes and thus elucidate the function of those genes (Figure 1A) [8]. Our current knowledge of PCa is owed to the numerous mouse knockout models made available through gene targeting.

Critical for the development of conditional knockout models is the site-specific recombinase technology known as Cre-Lox recombination (Figure 1B). This technology allows DNA modification—deletions, insertions, translocations, and inversions—at specific sites to be targeted to a particular cell type, or to be triggered by a particular external stimulus [9]. Derived from the bacteriophage P1, Cre recombinase is an enzyme that catalyzes a site-specific recombination event between two DNA recognition sites known as LoxP sites [9]. In 1994, the laboratories of Dr. Jamey Marth and Dr. Klaus Rajewsky reported that this system could be used for conditional gene targeting [9]. Since then, Cre-Lox recombination has been widely used to manipulate genes and chromosomes, creating genetic knock-out or knock-in mouse models [9]. In addition, the use of CRISPR (clustered regularly interspaced short palindromic repeats)/Cas 9 genome editing has further strengthened our understanding of the role of genes in cancer (Figure 1C). This technology can be designed to introduce RNA-directed, double strand DNA breaks or single strand ‘nicks’ using a mutated form of Cas9, a DNA endonuclease [10]. CRISPR is a simple and effective mechanism to introduce mutations both in vitro and in vivo that mimic somatic mutations, revolutionizing mouse models of cancer [10].

Thanks to DNA sequencing and gene expression data in the 21st century, PCa research models and our understanding of PCa as a whole have expanded tremendously. In 2002, The International Mouse Genome Sequencing Consortium, which was part of the Human Genome Project, achieved a high-quality draft sequence of the mouse genome [11]. Drafts for the Human Genome Project were already established a couple of years prior to the draft of the mouse genome, and the final sequence mapping of the human genome was finished in 2003 [11]. Although mice and humans are separated by 75 million years of evolution, their genomes are about 90% similar and both share almost 30 thousand genes [11]. Thanks to their similarities, the field known as comparative genomics can explore how mouse genes and their human counterparts contribute to health and disease. Consequently, the genes, mutations, and signaling cascades discovered from studying tumor progression in the prostates of lab mice could be applied to enhance our understanding of human pathophysiology.

The use of small animal-mammalian model such as mouse for cancer studies, and specifically here, for the prostate cancer studies is obvious due to relatively quick and easy breeding schemes, availability of genomic sequence information, ease of manipulation of embryonic cells in vitro, stability of genotype(s), and availability of multiple mouse strains with specific gene alterations [12,13]. These advantages weigh out some important anatomical and physiological differences between the mouse and human prostates [13]. Current mouse models of prostate cancer consist of two major types of models: xenografts/PDXs and genetically engineered mouse (GEM) models [14] (Figure 1, Table 1). The ease of xenograft studies such as short time from tumor implantation to tumor development and easy access to tumors (even by palpation in subcutaneous models), make xenograft models an attractive and critical step in many current preclinical drug tests [15]. The disadvantages of xenograft models comprise low availability of tumor cell lines with specific gene alterations, different tumor growth kinetics comparing to human tumors, lack of proper stromal-tumor interactions (although orthotopic models resembles the human situation better than subcutaneus tumor models in this regard), as well as lack of proper immunological responses in nude mice make the information obtained from xenograft models limited. In contrast, GEM models overcome most of the xenograft shortcomings. First of all, tissue-specific developmental (through the use of developmentally regulated tissue-specific promoters driving Cre recombinase expression [16,17], or conditional (through the use of tamoxifen-responsive promoter and retroviral vectors) disruption or overexpression of targeted genes resembles closely mutation-driven inactivation of human tumor suppressors or activation of oncogenes, respectively, in situ. This allows evaluation of the process of tumorigenesis from early time points of gene inactivation, through early histopathological changes, and subsequently through tumor growth and metastases if such occur. All of these can be studied in the context of physiological tumor-stromal interactions and the intact immune system. Further on, the possibility of evaluation of different levels of tumor suppressor inactivation (through one- or two-allele knockouts, or production of hypomorphic [18], as well knock-in mutant strains) allows formation of very specific hypotheses regarding both cell signaling pathways (that can be studied using primary cell isolated from GEM animals) as well as production of specific preclinical models [19,20]. These in turn will allow testing of specific drug formulations that may be then applied to phase I and II of clinical trials later on. Development of preclinical animal models for drug development is a critical step for development of better treatments for prostate cancer [15,21].

## 2. Patient-Derived Xenografts and Organoids of PCa

The shortage of clinically relevant, in vivo models is a large barrier to the understanding of tumor progression seen in PCa. Generating models that are derived from biopsy specimens and metastatic lesions from human patients is one means of mitigating this dilemma. One form of patient-derived models is xenografts (PDXs), which allow researchers to study and appreciate the tumor heterogeneity of prostatic diseases. It has been shown that populations of PDX mice upon passaging preserve most of the genetics of the original human tumors [22]. Extensive copy number alterations between human tumors and those in the PDX models were not found [22]. One type of PDX model, LuCaP PDXs, which harbor genetic alterations such as androgen receptor amplification, PTEN deletion, and TP53 deletion, demonstrate molecular heterogeneity in response to androgen deprivation therapy (ADT) and docetaxel treatment [23]. In PDXs studying hormone-naive prostate cancer, the Growth Factor Receptor Bound Protein 10 (GRB10) gene was found to be the most significantly upregulated, showing increased expression during and before the development of castration resistant prostate cancer [24]. Furthermore, PDXs were also established that are based on subrenal capsule grafting of patients’ tumor tissue into nonobese diabetic/severe combined immunodeficient (NOD/SCID) mice [25]. These PDXs preserve crucial properties of the original malignancies, including histopathological and molecular characteristics, reactions to androgen suppression, and tumor heterogeneity [25]. Consequently, PDXs represent valuable models not only for comprehending prostate tumor progression but also establishing drug screenings and therapies [25].

Despite their advantages, xenograft models are costly, time consuming, and require the use of immunocompromised mice. Three-dimensional (3D) patient derived organoids (PDOs) are perhaps a more efficient alternative—one that still maintains tumor heterogeneity and appropriate disease modeling. Organoids, or “mini-organs”, are clusters of cells grown in vitro that self-organize and differentiate into functional cell types [26]. Like PDXs, PDOs can be derived from primary tissue materials such as needle biopsies [26], but it has also been demonstrated that they can be derived from PDXs themselves [27]. These PDOs can conserve the genotypic and phenotypic characteristics of the LuCaP PDX cells they are derived from and represent an effective model to study mechanisms of resistance to ADT in PCa [27]. Organoids can also be derived from stem cells, including those expressing Lgr5, which is a genetic marker of Wnt-dependent stem cells found in tissues including the prostate [28]. In addition, organoids have also been used to study uncommon variants of PCa, including neuroendocrine prostate cancer (NEPC) derived from four human patients [29]. In these PDOs, the epigenetic modifier histone methyltransferase enhancer of zeste 2 (EZH2) was found to show increased expression in NEPC compared to adenocarcinomas [29]. Overall, PDOs share a similar mutational landscape with PCa, and they recapitulate in situ histology both in vitro and in vivo [30].

## 3. Comparing and Contrasting Types of PCa Models

**Figure 2:**
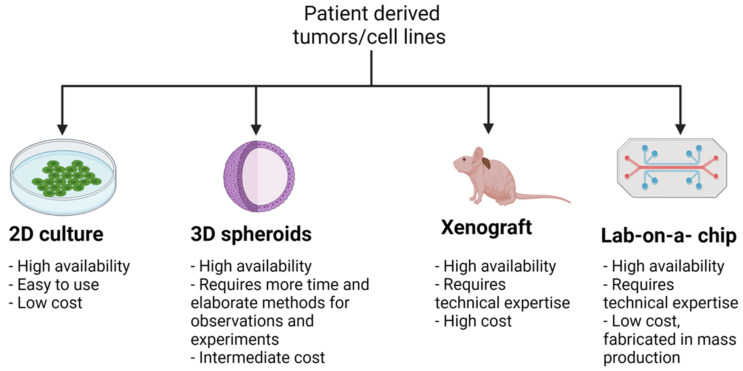
Summary of patient derived tumor models and cell lines. Various models of cancer are in use, each with their own applications, advantages, and disadvantages. 2D cultures are a widely used model for understanding cell biology and tissue morphology, as well as mechanisms of disease progression and actions of drugs [31]. Their high availability, ease of use, and low cost allow for high throughput data collection, although data from 2D cell lines alone is usually insufficient without further studies that more closely mimic in vivo conditions. 3D models, such as organoids and spheroids, overcome the 2D model’s limitation of monolayer cell culture, and thus obtain a more accurate physicochemical environment that compares to in vivo conditions [32]. This comes with the disadvantage of requiring more time and more elaborate methods for observations and experiments compared to 2D cultures, as well as a higher cost. However, 3D models do not require the same level of time and technical expertise as xenograft models, nor do they require the use of immunocompromised mice. The major advantage of xenograft models is that, compared to 2D and 3D models, they are the most comparable to in vivo physiology, permitting more accurate analysis of molecular, mechanistic, and drug screening studies. Lastly, lab-on-a-chip models are novel devices that integrate laboratory studies onto a very small, single, integrated circuit, allowing for high throughput screening [33]. As they are fabricated in mass production [34], lab-on-a-chip models have a high availability and low cost. Tumor-on-a-chip or organ-on-a-chip models are currently being developed to perform drug screening studies and better emulate physiologic conditions [35]. Created with BioRender.com.

The most common models used to study PCa in the lab include 2D cell lines, 3D organoids and spheroids, xenografts and allografts, PDXs, and genetically engineered (GE) mice, which are summarized in Table 1. GE mice include those with transgene expression and knockouts; knockouts may either be conditional or constitutional knockouts. The availability, ease of use, and cost of 2D cell lines and different patient derived tumor models, including novel lab-on-a-chip models, are provided in Figure 2.

## 4. Mouse Models Based on Tumor Stage Progression

This section highlights the pathological stages of prostate cancer that mouse models can represent. Significant non-neoplastic and neoplastic changes to the mouse prostate epithelium are outlined, and the genetic lab models associated with each stage of tumor development are included. For a more expansive review of PCa progression, please see Shappell et al. [13], which outlines prostate pathological changes in greater detail, including changes made to the prostate epithelia as well as the stroma.

### 4.1. Hyperplasia

Hyperplasia (Table 2) is defined as the proliferation of normal cells, and it is a non-neoplastic change to the prostate epithelium. Epithelial hyperplasia in the mouse can be diffuse or focal, and with or without small areas of nuclear atypia [13].

### 4.2. Prostatic Intraepithelial Neoplasia

Mouse prostatic intraepithelial neoplasia (mPIN) (Table 3) is the focal proliferation of atypical cells, contained within the gland or duct [41]. mPIN is neither benign nor malignant, but is described instead as a neoplastic proliferation of premalignant potential [13]. mPIN appears to be the consequence of a clonal expansion of a single transformed cell [41]. mPIN can be subclassified as (1) mPIN with documented potential to invasive carcinoma or (2) mPIN without documented potential to invasive carcinoma [13]. Human PIN, however, can be subclassified as either low grade or high-grade PIN (HGPIN); increased nuclear size and more prominent nucleoli (macronucleoli) are characteristic of HGPIN [13].

### 4.3. Microinvasive Carcinoma

Microinvasive carcinoma (Table 4) is the earliest recognizable form of invasive carcinoma, with penetration of malignant cells through the basement membrane of PIN-involved glands into the surrounding stroma [13]. Microinvasive carcinoma differs from invasive carcinoma by the greater extent of infiltration and destructive growth in the latter [13].

### 4.4. Invasive Carcinoma

Invasive carcinoma is described as the invasion of atypical, or malignant, epithelial cells into the surrounding stroma [32]. Most often, these epithelial cells are the luminal cells, and the increase in luminal cells is accompanied by a loss of basal cells [32]. The neoplastic growth pattern characterized by invasive carcinoma is incompatible with architecturally normal glands, and the greater extent of invasion in invasive carcinoma separates it from microinvasive carcinoma [26].

#### 4.4.1. Adenocarcinoma

Most invasive carcinomas in human PCa are adenocarcinomas (Table 5). Many epithelia contain specialized cells that secrete substances into the ducts or cavities that they line. In the prostate, these epithelial cells are termed the luminal cells, and prostate adenocarcinomas are derived from malignant luminal cells. Adenocarcinomas are subclassified as well, moderately, or poorly differentiated, according to the extent of glandular formation [26]. Discrete, well-formed glands are predominantly or exclusively observed in well-differentiated prostate adenocarcinomas, whereas solid sheets or nests are predominantly or exclusively observed in poorly-differentiated adenocarcinomas [13].

#### 4.4.2. Squamous Cell Carcinoma

Some epithelial sheets serve to seal the cavity that they line and to protect the underlying cell populations. Tumors that arise from epithelial cells forming these protective cell layers are termed squamous cell carcinomas (Table 6). In the prostate, this is seen in the form of keratinization and/or intercellular bridges [13].

#### 4.4.3. Neuroendocrine Carcinoma

Neuroendocrine (NE) carcinomas (Table 6) are characterized by the invasion of atypical neuroendocrine cells into the surrounding stroma. NE cells have traits of both nerve cells and hormone-producing endocrine cells, and they are intraepithelial regulatory cells that secrete a variety of neurotransmitters and peptide hormones that play a role in the growth and development of the prostate gland [78]. NE carcinomas do not exhibit well-defined glandular formation or extensive secretory differentiation [13].

**Table 6:** PCa models in which squamous cell carcinoma, neuroendocrine carcinoma, and sarcomatoid carcinoma are documented.

Model	Alteration	Driver and/or Add. Genetic Alteration	Phenotype	Reference
*Pten^flox/flox^*	Loss of expression	Nkx3.1-CreER^T2^ driver	Microinvasive AD with areas of poorly differentiated AD; squamous metaplasia	[79,80]
*RAR* *γ*	Loss of expression	C57BL/6 F1 background strain	Squamous metaplasia in prostate and seminal vesicles	[81]
*MYCN*	Gain of expression	Homozygous loss of *Pten* (conditional *Pten* allele)	Invasive adenocarcinoma with neuroendocrine PCa (NEPC)	[82]
*TRAMP*	Gain of expression	PB promoter driving expression of SV40 early region	Androgen independent tumors are 100% synaptophysin positive, and metastases are 67% positive	[83]
*LADY*	Gain of expression	Large PB (LPB) promoter driving expression of SV40 large T-antigen (Tag)	Visceral metastasis; NEPC	[84]
*LADY*	Gain of expression	LPB driver, 12T-7s line; crossed with PB-Hepsin	Adenocarcinoma with neuroendocrine differentiation (NED); NE metastasis to liver, lung, and bone	[85]
*LADY*	Gain of expression	LPB driver, 12T-7s line; crossed with β-catenin	Adenocarcinoma with focal NED, but without apparent NEPC	[86]
*Kras G12D*	Gain of expression	Homozygous loss of *Pten* (conditional *Pten* allele)	Invasive adenocarcinoma, sarcomatoid differentiation, with extensive metastasis	[87,88]
*Pten* and *p53*	Loss of expression	PB-Cre mediated deletion of *Pten* and *Trp53*; activation of ROSA-LSL luciferase reporter	Fast-growing, lethal sarcomatoid tumors; local invasive PCa	[89]
*ALK* and *N-myc*	Gain of expression	FVB/NJ and NSG background strains	Neuroblastoma development; metastasis with NED	[90]

#### 4.4.4. Sarcomatoid Carcinoma

Sarcomatoid carcinomas, or spindle cell carcinomas (Table 6), are derived from atypical spindle cells [41]. Sarcomatoid is a term that means ‘resembling sarcoma’. Sarcomas are tumors that are not of epithelial origin but rather of mesenchymal origin—connective tissue of the body such as stromal cells [41]. Sarcomatoid carcinomas arise from epithelial origin but resemble sarcomas, containing both epithelial and mesenchymal pathological properties.

### 4.5. Metastasis

Metastasis (Table 7) refers to the spread of cancer cells from the place where they first formed, or the primary tumor mass, to another part of the body through blood or lymphatic vessels [13]. Once the cells have reached a new organ or tissue, they undergo the process of colonization, or the growth of a micrometastasis into a macrometastasis. In mouse prostate cancer, metastasis usually occurs in the lymph nodes, visceral organs such as the lungs, and very rarely bones [41].

## 5. Mouse Models Based on Signaling Pathways

This section highlights the various signaling pathways characteristic of PCa, illustrating how PCa mouse models represent what happens in the context of human cancers. For each signaling pathway, the major players, or genes, that are affected are included, as well as the specific models that study those particular genes. Although the number of signaling pathways disrupted in PCa is essentially innumerable, the six below have been chosen to be investigated due to their significance in PCa pathology.

### 5.1. AR Pathway

Almost all prostate cancers express the AR (androgen receptor), which is a nuclear receptor that binds to androgens such as testosterone and dihydrotestosterone [110]. Upon binding androgens in the cytoplasm, ARs translocate to the nucleus and undergo dimerization; the dimer then acts as a transcription factor by binding to sequences of DNA called hormone response elements [111]. ARs may also interact with other proteins, causing upregulation or downregulation of specific genes, as necessary for the maintenance and development of the prostate [111].

Examples of genes that are involved in the AR pathway include the Erg and Etv1 genes, which are both members of the ETS (erythroblast transformation-specific) family of transcription factors [67]. Conditional gain of expression of Erg results in foci of invasive adenocarcinoma with varying levels of Erg expression [67,68], whereas conditional gain of expression of Etv1 causes invasive adenocarcinoma with homogenous Etv1 expression [69]. Both of these models are proposed to be caused by enhanced AR signaling [67,68,69]. This model also includes homozygous loss of the conditional Pten allele; the protein product of the tumor suppressor gene *Pten* acts as a phosphatase in the regulation of the cell cycle.

Upon the gain of expression of the SPOP F133V gene, adenocarcinoma that is invasive, poorly differentiated, and highly proliferative is observed due to SPOP mutation-induced AR signaling activation [71]. It is established that missense mutations in SPOP are the most common point mutations in primary prostate cancer [71]. Interesting to note is that in this model, SPOP-mediated AR activation is maintained against PI3K/mTOR mediated negative feedback [71]. Thus, the phenotype of the SPOP model is likely to be explained by both AR activation and PI3K activation. This model also includes homozygous loss of the conditional Pten allele.

In a different model, with the gain of expression of the MYCN gene and homozygous loss of Pten function, repression of AR signaling is cited as the proposed mechanism for promoting invasive adenocarcinoma with neuroendocrine prostate cancer (NEPC) [82]. The protein products of the proto-oncogene Myc family function as growth-promoting transcription factors in the cell nucleus. In this model, it was discovered that N-Myc binds to AR enhancers, and forms an interaction with the AR that is dependent on EZH2, an enzyme that participates in histone methylation and thus transcriptional repression [82]. It is believed that N-Myc and EZH2 cooperation shuts down AR signaling in the face of AR-directed therapy [82], thus promoting castration-resistant prostate cancer (CRPC).

### 5.2. PI3K Pathway

Alterations involving genes within the PI3K (phosphatidylinositol 3-kinase) signaling pathway are regularly observed in PCa [112]. The Ras family of small GTPases comprises proteins well known for their oncogenic involvement by setting in motion a variety of effector proliferative pathways—one of these pathways is the PI3K pathway [113]. Upon phosphorylation and activation by activated Ras protein, PI3K phosphorylates PIP2 (phosphatidylinositol (4,5)-diphosphate) into PIP3 (phosphatidylinositol (3,4,5)-trisphosphate). This in turn leads to the tethering of Akt (also known as PKB) and Rho-GEFs to the plasma membrane. Akt/PKB subsequently phosphorylates and activates effector proteins such as FOXO and mTOR, while phosphorylating and inactivating effector proteins such as GSK-3β and Bad [114]. Ultimately, this results in increased cell growth, cell proliferation, cell motility, cell survival, and protein synthesis [115]. One key player in this signaling pathway is Pten (Phosphatase and Tensin Homolog), a protein phosphatase that reverses the actions of PI3K by converting PIP3 into PIP2. In human prostate cancer, mutations and inactivation of Pten are more common than amplifications and activation of PIK3CA, PIK3CB, PIK3R1, and AKT1 [116].

Several GEMMs associated with the activation or inactivation of the PI3K signaling pathway are present in the PCa literature. As a matter of fact, the AR and PI3K signaling pathways are the two most frequently altered in localized and metastatic PCa [117]. One Nkx3.1 knockout model, with hemizygous loss of the germline Pten allele, features hemizygous or homozygous loss of the Nkx3.1 gene [55,56], which is an androgen-regulated, homeobox gene with tumor suppressive effects [37]. In these models, mice greater than one year of age develop mPIN and invasive adenocarcinoma, and sometimes metastasis to the lymph nodes [55,56]. Unlike other classical tumor suppressor genes, Nkx3.1 is unique in that it is inactivated through loss of protein expression, rather than through inactivation through mutation [118,119].

The NCoA2 and NSD2 knockout models are also understood to be mediated by the PI3K signaling pathway. The NCoA2 model features invasive adenocarcinoma with metastasis to the lungs and lymph nodes. The model is characterized by the conditional gain of expression of the gene that codes for NCoA2 (Nuclear Receptor Coactivator 2) [93]. NCoA2 is a transcriptional coregulatory protein that contains many nuclear receptor interacting domains as well as histone acetyltransferase activity, permitting un-inhibited gene expression and the activation of PI3K signaling [120]. The NSD2 model features the conditional gain of expression of the gene that codes for NSD2 (Nuclear Receptor Binding SET Domain Protein 2), also known as WHSC1 (Wolf-Hirschhorn syndrome candidate 1) [94]. Invasive adenocarcinoma with metastasis to the lymph nodes, lungs, and bone are found in this model [94]. Unlike NCoA2, NSD2 takes a different approach in stimulating the PI3K pathway. Higher levels of NSD2 transcriptionally upregulate expression of RICTOR, a vital constituent of mTOR complex 2 (mTORC2), to further enhance AKT/PKB activity [94]. Both the NCoA2 and NSD2 models are accompanied by the homozygous loss of the conditional Pten allele.

In addition to these models, the mpAkt model, which features gain of expression of Akt and Myc genes, as well as loss of Pten function, demonstrates accelerated progression of mPIN to microinvasive carcinoma [52]. Disruption of basement membrane integrity, stromal remodeling, and infiltration of immune cells are found in this model [52]. In particular, infiltration of B-lymphocytes, T-lymphocytes, and macrophages is noted in the early development of mPIN, continuing through the progression to microinvasive carcinoma for this model [52]. The inflammatory response mediated by the leukocytes found in the mpAkt model could prove of particular interest in elucidating the roles of the immune system in tumor growth regulation [52].

**Figure 3:**
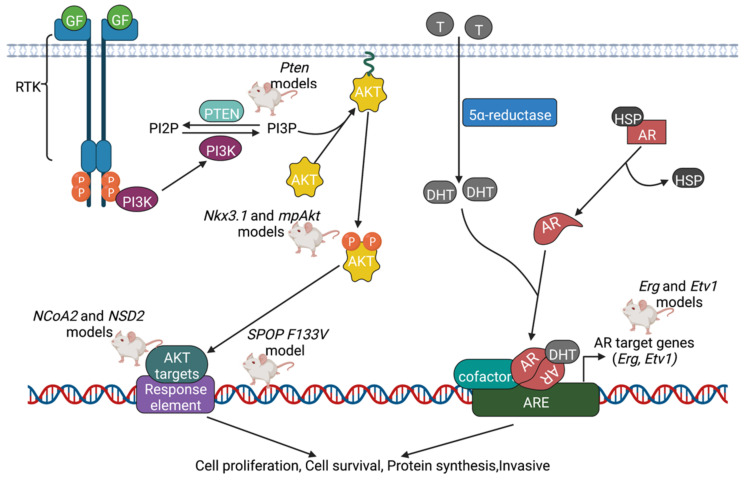
Summary of PCa knockout models driven by PI3K and AR signaling pathways. Several signaling pathways have been identified that play a role in the development of PCa. To explain the phenotypes of each KO model, researchers propose one or more signaling pathways that could be altered in the model that they developed. In the PI3K pathway, once activated by Ras protein, PI3K phosphorylates PIP2 into PIP3, leading to the tethering of Akt/PKB and Rho-GEFs to the plasma membrane. Pten reverses the actions of PI3K by converting PIP3 back into PIP2, and thus plays a key role in inhibiting cell growth and proliferation. In addition to the PI3K pathway, another oncogenic signaling pathway involves the nuclear receptor known as AR. In the presence of androgens such as testosterone and dihydrotestosterone, Heat Shock Protein (HSP) is released from AR, and AR binds the androgen in the cytoplasm. ARs translocate to the nucleus and undergo dimerization; the dimer then acts as a transcription factor by binding to androgen response elements (AREs). With recruitment of other cofactors, this again leads to increased cell proliferation, cell survival, and protein synthesis. Created with BioRender.com.

PCa knockout models driven by AR and PI3K signaling pathways are displayed in Figure 3.

### 5.3. TP53 Pathway

TP53 (Tumor protein 53), commonly referred to as the guardian of the genome, is a tumor suppressor responsible for controlling processes such as DNA repair, cell cycle (growth) arrest, and apoptosis [121]. In humans, the TP53 protein is coded by the TP53 gene; in mice, however, the protein is known as TRP53 and is coded by the TRP53 gene. Signals from metabolic stress or genomic damage, including a lack of nucleotides, UV radiation, ionizing radiation, oncogene signaling, hypoxia, and blockage of transcription cause TP53 levels to rapidly accumulate from its normally low steady state concentration [114]. TP53 regulates whether the DNA will be repaired, or if the damaged cell will undergo apoptosis. By preventing cells with mutated DNA from proliferating, TP53 plays a significant role in prohibiting the development of tumors [121]. As a matter of fact, TP53 is the most commonly mutated gene in human cancer [114].

In undisturbed cells, TP53 binds to the promoter of the Mdm2 (Mouse double minutes 2) gene, and the encoded Mdm2 protein binds to TP53 itself and initiates TP53′s ubiquitylation, export to cytoplasm, and degradation in the proteosome [114]. TP53 is protected from Mdm2 by being activated by the Ser/Thr-kinase Chk2; Chk2 is in turn activated by ATM, another Ser/Thr-kinase that receives signals from sensors of double-stranded DNA breaks [114]. TP53 is also protected from Mdm2 by being activated by the Ser/Thr-kinase Chk1; Chk1 is in turn activated by ATR, another Ser/Thr-kinase that receives signals from sensors of single-stranded DNA [114]. Likewise, ATM kinase can phosphorylate Mdm2, in a way that leads to its destabilization and thus promote increased levels of TP53 [114]. Another protein, ARF (p19ARF in mouse cells and p14ARF in human cells), can accumulate in the nucleolus and form stable complexes with Mdm2, and thus inactivate the TP53 antagonist [114].

One TRP53 mouse model features conditional loss of expression of the TRP53 gene [95], and consequently, invasive adenocarcinoma with metastasis to the lymph nodes, spleen, liver, and organs near genitourinary tract excluding the bladder [96] is observed. Cited signaling pathways include senescence bypass, Myc activation, and neuroendocrine differentiation [97]. However, homozygous loss of Pten function is also a part of this model, as Pten and TRP53 are often co-mutated in human castration resistant prostate cancer (CRPC) [97]. Conditional inactivation of TRP53 alone in the mouse prostate failed to produce a tumor phenotype [95]. Combined complete TRP53 and Pten inactivation was necessary to achieve invasive PCa, which was observed as early as 2 weeks post-puberty, and was consistently lethal by 7 months of age [95].

In addition, DNA damage signaling and genomic instability have been proposed in Chk1 knockout mice [64]. The Chk1 model features hemizygous loss of the Chk1 gene [64] which, as described above, encodes a Ser/Thr-kinase that phosphorylates/activates TP53 and phosphorylates/inactivates Mdm2. This model also features hemizygous loss of Pten function; the Chk1 and Pten compound haploinsufficiency promotes progression of HGPIN to invasive adenocarcinoma [64]. Interestingly, Chk1 was also proposed to be a direct transcriptional target of both Erg and Etv1 oncogenic activity [64]. Erg, for example, binds and transcriptionally represses the Chk1 promoter [64]. Therefore, the phenotype of this model may also perhaps be explained by the upregulation of AR signaling.

The Phlpp1 knockout model is characterized by the hemizygous or homozygous loss of the Phlpp1 gene, and the resulting phenotype is invasive adenocarcinoma, at full penetrance with onset of 8 months [63]. Phlpp1 loss alone results only in neoplasia; partial Pten loss is necessary for the development of invasive prostate cancer [63]. Phlpp1 (PH domain and Leucine rich repeat Protein Phosphatase 1), together with Phlpp2, are protein phosphatases that function as tumor suppressors by negatively regulating Akt/PKB [122], the kinase that integral to PI3K pathway described earlier. Akt/PKB is significant in the TP53 pathway because Akt/PKB phosphorylates and activates Mdm2 [114], which itself binds to TP53 and inactivates it by targeting it for degradation.

Lastly, in the novel ACSS3 model, increased expression of ACSS3 (Acyl-CoA synthetase short-chain family member 3) was found to limit PCa progression [49]. mPIN in the anterior zone of the prostate, as well as increased proliferation, migration, and invasion, are observed in response to loss of ACSS3 expression [49]. ACSS3 normally promotes endoplasmic reticulum (ER) stress, which in turn activates apoptosis [49], one of several pathways regulated by TP53. ACSS3 is postulated to downregulate a lipid-droplet coat protein called PLIN3 (also known as Perilipin 3 or Mannose-6-Phosphate Receptor Binding Protein) [49]. Tumor cells often show abnormal lipid accumulation in the form of lipid droplets, and ACSS3 inhibits such accumulation through its inhibitory effects on PLIN3 [49]. Thus, the ACSS3 model demonstrates a unique approach in the search for therapeutic perspectives for CRPC that have become resistant to endocrine therapy.

### 5.4. DNA Repair Pathway

In response to the presence of nucleotides of abnormal chemical structure, an intricate DNA repair pathway will be mobilized to repair the damage and maintain integrity of the genome [114]. Many of the pathways of DNA repair machinery are downstream of and directly induced by TP53, since TP53 levels accumulate upon signals such as a lack of nucleotides, UV radiation, ionizing radiation, and blockage of transcription [114]. One component of the DNA repair pathway is MGMT (O6-methylguanine-DNA methyltransferase), or also known as AGT (O6-alkylguanine-DNA alkyltransferase). This enzyme removes methyl and ethyl adducts from the O6 position of guanine [123]; this is in fact the only system of removing alkylated bases in human cells [114]. Two very important systems associated with repair machinery for single stranded DNA breaks include BER (base excision repair) and NER (nucleotide excision repair). BER enzymes cleave the glycosylic bond linking the modified nitrogenous base to the deoxyribose sugar, and act in response to lesions that do not create structural distortions of the DNA double helix [124]. These lesions are due to endogenous sources, such as reactive oxygen species and depurination events [124]. NER enzymes, however, cleave the entire modified nucleotide from the DNA helix, and act in response to lesions that create large, helix-distorting alterations [125]. These lesions are due to exogenous sources, such as UV photons and chemical carcinogens [125].

The BRCA1 and BRCA2 proteins are perhaps the most well-known constituents of the DNA repair pathway. These proteins gather a group of other DNA repair proteins, such as RAD50/Mre11 and Rad51, into large physical complexes to repair double-stranded DNA breaks [114]. Two separate mechanisms of repairing dsDNA breaks are homology-directed repair (HDR) and non-homologous end joining (NHEJ) [114]. HDR involves the presence of sister chromatids and thus is more accurate than the more error-prone NHEJ [114].

Due to the association between the TP53 pathway and DNA repair pathway in the cell, knockout models featuring altered DNA repair pathways are often presented with genomic instability: an outcome frequently observed in TP53 models. As described in the TP53 section, the Chk1 knockout model is characterized by DNA damage signaling and genomic instability upon hemizygous loss of Chk1 and Pten, and the resulting phenotype is invasive adenocarcinoma [64]. This model also includes hemizygous loss of Pten [64].

The Bmi1 model is driven by the gain of expression of a core component of the polycomb repressive complex 1, Bmi1 [61]. Conditional overexpression of Bmi1 alone results in mPIN, but when combined with hemizygous loss of the Pten, invasive PCa is generated [61]. Bmi1 overexpression with Pten haploinsufficiency specifically results in a locally invasive and highly vascularized adenocarcinoma, with frequent bladder outlet obstruction [61]. Akt/PKB was found to phosphorylate and activate Bmi1, which contributes to the modulation of the DNA damage response that tips in favor of genomic instability and oncogenic potential [61]. The phosphorylation of Bmi1 by Akt/PKB results in stimulated ubiquitination of the ubiquitin ligase known as histone 2A (H2A) at DNA double-strand breaks (DSBs), and thus defective DSB-promoted homologous recombination repair [61].

In the HoxB13/Myc model, invasive adenocarcinoma with metastases to the liver, lymph nodes, and lungs is observed [104]. Here, Myc overexpression as well as Pten loss are driven by androgen-independent Hoxb13 control elements in mouse prostate luminal cells [104]. This caused genomic instability and highly penetrant carcinoma with metastasis, in the absence of induced dysfunction of telomeres or interestingly in the loss of function of TP53 [104]. The gain of Myc expression alone or the loss of Pten function alone only resulted in mPIN [104]. HoxB13 itself encodes a transcription factor that is part of the homeobox gene family and functions as a tumor suppressor [126]. The G84E mutation in the HOXB13 gene has been firmly associated with an increased risk for familial PCa [127], and this model represents a valuable tool for representing the phenotypic effects of genomic instability without the loss of function to TP53.

**Figure 4:**
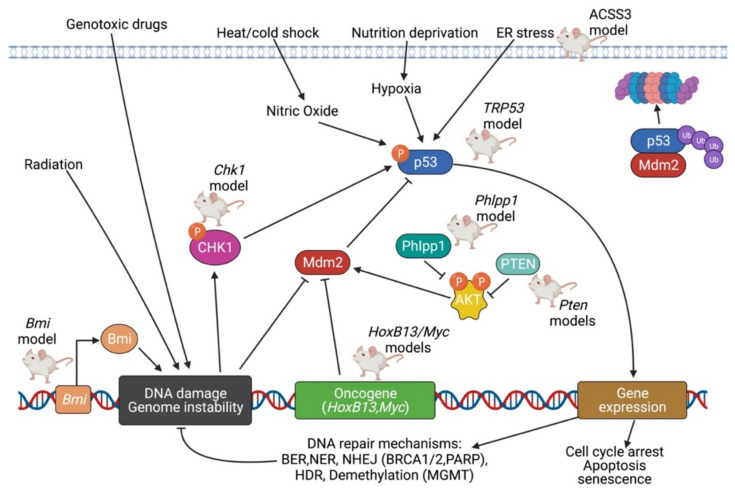
Summary of PCa knockout models due to inhibition of TP53 and DNA repair signaling pathways. In normal conditions, TP53 levels are at their low steady-state concentration. In the presence of metabolic stress or genomic damage—such as a lack of nucleotides, presence of UV radiation, ionizing radiation, oncogene signaling, hypoxia, or blockage of transcription—TP53 levels rapidly accumulate. As the guardian of the genome, TP53 regulates processes such as DNA repair, cell cycle arrest, and apoptosis; TP53 itself is activated by Chk1 and inhibited by Mdm2. The DNA repair stimulated by TP53 can itself be identified as its own signaling pathway. One component of the DNA repair pathway includes MGMT/AGT, which removes methyl and ethyl adducts from the O6 position of guanine. Excision pathways such as nucleotide-excision repair and base-excision repair can be stimulated in response to single-stranded DNA breaks. With the help of proteins such as BRCA1 and BRCA2, double-stranded DNA breaks can be repaired via either homology-directed repair or non-homologous end joining. Created with BioRender.com.

PCa knockout models due to inhibition of TP53 and DNA repair signaling pathways are displayed in Figure 4.

### 5.5. MYC Pathway

The oncoproteins of the Myc family, when expressed in a deregulated fashion, function as growth-promoting transcription factors in the cell nucleus [114]. More than 50% of human cancers overexpress either Myc (often termed c-Myc), or one of its two close cousins: N-Myc and L-Myc [128]. These proteins are encoded by the C-MYC, MYCN, and MYCL genes, respectively [128]. Myc family proteins all share a homologous motif at the C-terminus that consists of a basic DNA-binding domain, followed by amino acid sequences forming an α-helix, a loop, a second α-helix, and a Leucine zipper (BR/HLH/LZ motif) [128]. Myc, as well as other transcription factors with this BR/HLH/LZ motif, form homodimers or heterodimers with themselves [114]. The dimer can associate with specific regulatory DNA sequences called E-boxes (CACGTG), found on the promoters of target genes [114]. The association between Myc and its partner Max, for example, drives the expression of a large cohort of genes that favor cell growth and proliferation [128]. Myc/Max is able to induce expression of Cyclin D2 and CDK4, which promote advance through early G1 phase of the cell cycle, as well as Cul1 and Cks1, which degrade p27^Kip1^, and thus, promote advance into the S-phase of the cell cycle [114]. Myc/Max also induces expression of the genes encoding the E2F transcription factor proteins, which are normally negatively regulated by pRb [114]. The activation of these target genes is made possible due to the recruitment of chromatin-modifying complexes such as GCN5, TIP60, TIP48, and TRRAP by Myc/Max [128].

One GEMM that is driven by Myc, and one that has already been described in the section describing the AR signaling pathway, is the MYCN model, which features conditional gain of expression of the MYCN gene and homozygous loss of Pten function [82]. In this model, repression of AR signaling is cited for causing invasive adenocarcinoma with neuroendocrine prostate cancer [82]. EZH2 is one of the transcription factors that N-Myc interacts with to co-repress transcriptional expression of target genes [82]. N-Myc and EZH2 cooperation was found to abrogate AR signaling irrespective of Pten status or the degree of prostate pathology [82]. Pten was chosen to be knocked out since Pten deletion is such a common characteristic of CRPC (50%) [116]. Thus, MYCN mice represent a valuable model for studying neuroendocrine prostate cancer that is resistant to androgen deprivation therapy.

In addition to the Nkx3.1 model explained in the section describing the PI3K signaling pathway, a different Nkx3.1 model features enhanced Myc transcriptional activity [53]. This model is characterized by the conditional loss of expression of the tumor suppressor Nkx3.1, in the presence of Myc transgene activation that is driven by the CMV enhancer and β-actin promoter [53]. It was strikingly found that several genes targeted by Nkx3.1 are also direct targets of Myc [53]. Thus, a disruption in the co-regulation of these target genes can result in prostate tumorigenesis. When Myc is overexpressed and Nkx3.1 is underexpressed, genes such as Nedd4l, Hk2, Clic4, and Igf1r are upregulated, while genes such as Prdx6, Ace, Mt2, Prkca, Ugcg, Ceacam1, Itpr2, Cflar, and Atf3 are downregulated [53]. Overall, Myc overexpression and Nkx3.1 underexpression results in HGPIN with microinvasion, likely mediated by these an imbalance in the various target genes shared by Myc and Nkx3.1 [53].

The Braf^V600E^ model is characterized by the gain of expression of the Braf gene as well as homozygous loss of Pten function. Braf encodes a protein known as B-Raf, which is a member of the Raf kinase family of growth signal transduction protein kinases [129]. Senescence bypass and invasive adenocarcinoma with metastasis to the lymph nodes, bone marrow, and lungs is observed in this model [105]. This model demonstrates that inducible expression of Braf activates Erk 1/2 signaling, and inducible loss of expression of Pten activates PI3K-Akt-mTOR signaling [105]. In addition to activated Ras protein stimulating PI3K, Ras protein also stimulates B-Raf and other Raf kinases [130]. Raf kinase phosphorylates and stimulates MEK (also known as MAPKK, or mitogen activated protein kinase kinase). MEK phosphorylates and stimulates Erk 1/2 (also known as MAPK, or mitogen activated protein kinase). Erk 1/2 can then stimulate kinases in the cytoplasm that regulate translation as well as transcription factors in the nucleus [130]. The PI3K-Akt-mTOR signaling and Erk 1/2 signaling pathways are coincident with Myc pathway activation in promoting advanced prostate cancer with metastasis [105].

As illustrated by these three models, there is a large overlap in the Myc, AR, PI3K, and Erk 1/2 signaling pathways—all of which are major oncogenic conduits leading to cell growth and proliferation. Furthermore, in the recent RIPK2 model, receptor-interacting protein kinase 2 (RIPK2) has been found to strongly regulate the stability and activity of c-Myc [109]. This is done through the association of RIPK2 with a form of MEK (MAPKK) called MAPKK7 [109]. Invasive adenocarcinoma with metastasis to bone is observed in this model. [109] Drugs that inactivate the noncanonical RIPK2/MEK/c-Myc pathway are proposed as a therapeutic target in impairing PCa metastasis [109].

### 5.6. Wnt Pathway

18% of a total 150 patients afflicted with mCRPC (metastatic castration-resistant prostate cancer) were found to harbor alterations in the Wnt signaling pathway in a genomic study [116]. The Wnt signaling pathway can be subclassified as canonical Wnt signaling or non-canonical Wnt signaling—both pathways involve Wnt growth factors that bind to a family of Frizzled receptors [131]. In canonical Wnt signaling, and in the absence of Wnt, a complex of Axin and APC allows GSK-3β (Glycogen Synthase Kinase-3β) to phosphorylate and target for destruction β-catenin [131]. In the presence of Wnt, however, GSK-3β is inhibited, allowing β-catenin to avoid destruction, accumulate, and enter the nucleus [113]. There, β-catenin associates with a group of DNA-binding proteins termed Tcf/Lef, and such protein complexes enable expression of genes that favor proliferation and the stem cell state [114]. In individuals with mCRPC who harbored alterations in the Wnt signaling pathway, hotspot activating mutations in the gene that codes for β-catenin, CTNNB1, were observed [116]. Recurrent alterations were also seen in the tumor suppressor gene known as APC (adenomatous polyposis coli) [116], which plays a critical role in negatively regulating β-catenin levels. By promoting increased differentiation and decreased proliferation, APC prevents formation of an adenomatous polyp or tumor [114].

GEMMs associated with altered Wnt signaling have been relatively understudied compared to models based on other signaling pathways [132]. However, the role of the Wnt signaling pathway should not be underestimated, for it has been validated as a therapeutic target in metastatic prostate cancer [133]. In knockout mice harboring probasin-Cre-mediated deletion of Apc, hyperplasia is noted as early as 4.5 weeks of age, and adenocarcinoma is noted by 7 months [60]. Subsequent to the loss of Apc, levels of β-catenin protein become elevated, allowing for highly proliferative cells and ultimately prostate carcinogenesis. No metastases to sites such as the lymph nodes or other glands were observed [60]. Significant portions of carcinoma remained for 2 months after castration, and therefore, tumors harboring Apc loss of expression can grow under conditions of androgen depletion [60]. This illuminates the importance for the search for non-androgen therapies in invasive Pca.

One transgenic model features gain of expression of β-catenin in mice that also express the large probasin promoter directed SV40-Large T-antigen (LPB-Tag) [86]. Mice expressing nuclear β-catenin alone and those expressing Tag alone were only able to reach mPIN, whereas the combination of the two results in invasive adenocarcinoma [86]. It was discovered that prostates of these genetically altered mice exhibit focal areas of neuroendocrine differentiation (NED), but NEPC does not develop [86]. The finding that β-catenin induces NED was confirmed using an in vitro study that involved expression of a non-degradable β-catenin gene in T-antigen expressing NeoTag1 cells, a prostatic epithelial cell line derived from the 12T-7f mouse line [86]. Active Wnt/β-catenin signaling was able to induce increased expression of Foxa2, a forkhead transcription factor, as well as two other well-established neuroendocrine markers: NSE (neuron specific enolase) and Chromogranin A [86]. Furthermore, it was found that in vivo, increased Wnt/β-catenin signaling downregulated both AR and AR signaling, a feature correlated with NED [134].

In a different model, co-activation of ALK and N-Myc is able to transform mouse prostate basal stem cells into aggressive PCa with neuroendocrine differentiation [90]. ALK (Anaplastic lymphoma kinase) is a receptor tyrosine kinase that plays a role with N-Myc in the pathogenesis of neuroblastoma and other malignancies, including those of the prostate [90]. ALK/N-Myc tumors display activation of the Wnt/β-catenin signaling pathway. If ALK is stimulated, Wnt is also stimulated, leading to the development of NEPC and neuroblastoma in vitro, and tumor growth and metastasis in vivo [90]. Therefore, this model illustrates a unique protein in ALK as a novel factor that can cooperate with N-Myc to promote the development of NEPC [90].

Lastly, in the MMP7 model, invasive adenocarcinoma through induction of epithelial-to-mesenchymal transition (EMT) is observed [75]. MMP7 (Matrix Metalloproteinase 7) is a target gene of Wnt/β-catenin that is responsible for catalyzing destruction of the extracellular matrix. It was found that the pro-inflammatory cytokine IL-17 induces MMP7 expression to release β-catenin from the E-cadherin/β-catenin complex [75]. Released β-catenin was able to promote EMT, in which epithelial cells lining the prostatic glands decrease expression of epithelial markers such as E-cadherin, claudin, and zona occludens 1, and increase expression of mesenchymal markers such as N-cadherin and vimentin [75]. This permits tumor cells to break through the basement membrane of the surrounding gland and promote invasion and eventually metastasis.

**Figure 5:**
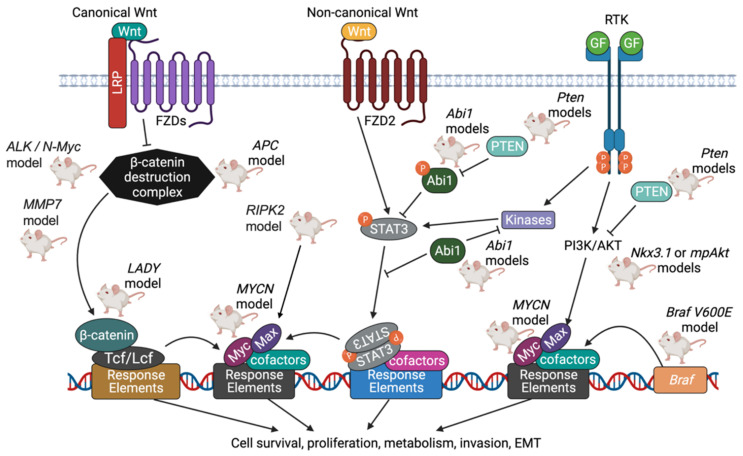
Summary of PCa knockout models driven by MYC and Wnt signaling pathways. Myc family oncoproteins, including c-Myc, N-Myc, and L-Myc, function as growth-promoting transcription factors when expressed in a deregulated fashion. Myc proteins form homodimers with themselves, or as heterodimers with other proteins that share similar structures, before associating with specific DNA response elements. Max, whose levels are increased through the actions of the PI3K pathway, is one protein that associates with Myc. Together, Myc and Max promote advance through the cell cycle by stimulating Cyclin D2/CDK4 and inhibiting p27^Kip1^. In canonical Wnt signaling, Wnt binds to the Frizzled receptor family, and the β-catenin destruction complex (which includes Axin, APC, and GSK-3β) is inhibited. The levels of β-catenin accumulate, and β-catenin complexes with DNA binding proteins termed Tcf/Lef, which together enable expression of genes that favor cell survival and proliferation. In non-canonical Wnt signaling, STAT3 (signal transducer and activator of transcription 3) is activated in response to the binding of Wnt to Frizzled receptors. Proteins such as Abi1 inhibit STAT3 from entering the nucleus and acting as an oncogenic transcription factor [135]. Created with BioRender.com.

PCa knockout models driven by MYC and Wnt signaling pathways are displayed in Figure 5.

## 6. Future Directions

There are two areas of evident use for mouse models of PCa. Xenografts and PDXs have already been extensively used for preclinical studies. Testing novel drugs and treatment approaches, represents the most direct use for these models to provide critical information leading to clinical trials. Selection of the specific cell line/PDX depends on the activation/presence of drug target/pathway in the model [136,137]. The knowledge of genomic sequence and gene expression for the cell lines in use is critical for proper evaluation of the prospective drug use [132].

The “co-clinical trial” was the concept introduced about the decade ago. It was based on success of APL paradigm that led to successful therapy based on the information gained from the model mouse of the disease (provided the changes parallel the human pathology), which led to optimized treatment of APL [138]. The concept proposes to use GEM models in parallel to human phase I/II trials to speed up the treatment development. What GEM models offer is well-defined system for rapid evaluation of treatment effect on disease progression, biomarker response and development of potential drug resistance pathways [139]. This information can then be transferred to the clinic and tested in human patients. Here, specific PDX models may be available for subsequent precision medicine development [97,140].

The use of GEMs for immunological application is growing, with two applications available: gene interactions within tumors may inform about specific tumor immunogenicity and effects on tumor environment [141,142]. The genes can be also manipulated in the microenvironment and then examined using GEMs. This possibility provides the advantage of GEMs over other model systems.

## 7. Conclusions

Our knowledge of PCa has greatly increased thanks to the diverse array of models that have been established over the past several decades. Following the development of the first immortalized cell lines, more sophisticated technologies such as gene targeting using embryonic stem cells, Cre-Lox recombination, and CRISPR/Cas9 gene editing allowed for a more comprehensive and accurate understanding of human pathophysiology. The models most commonly used today to study tumorigenesis, including 2D cultures, 3D organoids and spheroids, xenografts and allografts, and knockout mice, have their unique applications, advantages, and disadvantages. After choosing a model, the researcher must decide which gene(s) of interest to manipulate to define the pathway of tumor progression. The resulting phenotype—such as the stage of tumor progression and degree of metastasis—as well as the disrupted molecular signaling pathways elucidate the function of the studied gene in either promoting or inhibiting the progression of PCa. The signaling pathways most often identified in PCa models include AR, PI3K, TP53, Myc, Wnt, and DNA repair pathways. As novel models are being generated, bringing all the models together enables the researcher to identify clearer connections between the genes, stages of tumorigenesis, and signaling pathways linked with PCa. Moreover, these models provide a critical tool for drug sensitivity studies, an obligatory step in translational research.

## Figures and Tables

**Table 1:** Advantages and disadvantages of models for PCa studies.

Model	Availability, Ease of Use, Cost	Major Applications	Advantages	Disadvantages
2D Cell Lines	-High availability -Easy to use -Low cost	-Molecular and mechanistic studies -Drug screening studies -Validation experiments -Epigenetic studies	-Relatively quick results -Data may be made available online -Phenotypic analysis by microscopy studies	-Results may only apply to particular cell line, unless repeated in multiple cell lines -Lacks intra-tumor heterogeneity -Intrinsic effects due to high level passaging -Lacks phenotypic characteristics of parental tumors
3D Organoids, Spheroids	-High availability -Requires more time and elaborate methods for observations and experiments -Intermediate cost	-Molecular and mechanistic studies -Drug screening studies -Imaging and observational studies	-Data can be easily obtained in a relatively short time -Phenotypic analysis by microscopy studies -High content data such as drug screens -More relevant to human cancer -Easily accessible for DNA and mRNA sequence analysis -Presence of ECM	-Longer timescale than 2D cell lines -Requires more extensive analysis -Data obtained is dependent on environment, causing high variability
Xenografts, Allografts	-High availability -Technical expertise required to use -High cost	-Drug response studies and novel drug screening studies -Confirmation of molecular and mechanistic studies	-Comparable to in vivo context -Drug approval studies -Easily accessible for DNA and mRNA sequence analysis	-More time-consuming than 2D or 3D models -Drug response associated with genotype -Immunosuppression limits understanding the role of the immune system in tumor response (alleviated for allografts)
Patient Derived Xenografts (PDXs)	-Limited availability -Patient consent required to use -Technical expertise required to use -High cost	-Heterotopic injection or transplant -Understanding tumor heterogeneity -Personalized drug testing for effective therapy -Maintenance of tumor architecture -In vivo physiology	-Easily accessible for DNA and mRNA sequence analysis -Understanding of drug resistance response -May lead to specific identification for treatment target	-Subsequent confirmation studies more difficult as each model is unique to each patient -Immunosuppression limits understanding the role of the immune system in tumor response
Genetically Engineered (GE) Mice -With transgene expression -Knockouts (KOs) -Constitutive -Conditional	-Limited number of models -Animal colony maintenance required -High cost	-Establishment of in vivo functions of oncogenes and tumor suppressor genes -Genetic interactions -Tumor progression studies -Epithelial-stromal interactions -Immunological studies -Platform for studying metastasis	-Definitive functional studies, including metastatic potential -Establishment of in vivo functions in context of different tissues -Easily accessible for DNA and mRNA sequence analysis	-Long-term studies, with long tumor latency -Time and high cost associated with breeding skills and genotyping -Many common genotypes not represented -Patented strains unavailable -Phenotype may be influenced by strain -Spontaneous, strain-dependent tumorigenesis independent from genetic engineering

**Table 2:** PCa models in which hyperplasia is documented.

Model	Alteration	Driver and/or Add. Genetic Alteration	Phenotype	Reference
*p27^Kip1^* ^(1)^	Loss of expression	Created by gene targeting in embryonic stem cells	Hyperplasia of multiple organs, including prostate, testis, and thymus	[36]
*Nkx3.1*	Loss of expression	Genomic clones isolated from λFIXII library from 129Sv/J genomic DNA	Prostatic epithelial hyperplasia and dysplasia; decreased bulbourethral gland size	[37]
*Rb^flox^*	Loss of expression	PB^Cre4^ driver ^(2)^	Focal areas of epithelial hyperplasia; loss of basement membrane and smooth muscle layer integrity	[38]
*IGF-1^flox^*	Gain of expression	PB^Cre4^ driver ^(2)^	Cell autonomous proliferation; hyperplasia	[39]
*FOXA1*	Loss of expression	PB^Cre4^ driver ^(2)^	Progressive hyperplasia with extensive cribriform patterning	[40]

^(1)^*p27^Kip1^*: p27/kip1 is Cyclin-dependent kinase inhibitor 1B. ^(2)^*PB^Cre4^*: probasin promoter, a frequently used promoter used to direct transgene expression of Cre recombinase to prostate epithelial cells.

**Table 3:** PCa models in which PIN is documented.

Model	Alteration	Driver and/or Add. Genetic Alteration	Phenotype	Reference
*KDM5B*	Gain of expression	Loss of Pten function	HGPIN	[42]
*Sox9*	Gain of expression	Hemizygous loss of *Pten* (germline heterozygous *Pten* allele)	HGPIN	[43]
*Pten^flox/flox^*	Loss of expression	K14-Cre^ERT2^ driver ^(1)^	PIN development	[44]
*Pten*	Loss of expression	Mouse *Pten* disrupted by homologous recombination	PIN development; formation of aberrant embryoid bodies	[45]
*Pten x p53*	Loss of expression	Recombination of adult prostatic epithelium with embryonic rat seminal vesicle mesenchyme	HGPIN	[46]
*Abi1*	Loss of expression	PB^Cre4^ driver ^(2)^	PIN development	[47]
*EAF2*	Loss of expression	PB-CreER^T2^ driver ^(3)^	Luminal epithelial hyperplasia and mPIN	[48]
*ACSS3*	Loss of expression	Transfection of overexpressing lentivirus and sgRNA (CRISPR/Cas9)	PIN in anterior prostate; increased proliferation, migration, and invasion	[49]
*CSF-1*	Gain of expression	PB^Cre4^ driver	Immune cell infiltration into prostate; LGPIN	[50]

^(1)^*K14-Cre^ERT2^*: keratin 14 (*KRT14*) promoter driven Cre recombinase; ^(2)^*PB^Cre4^*: probasin promoter; ^(3)^*PB-CreER^T2^*: tamoxifen-inducible Cre under probasin promoter.

**Table 4:** PCa models in which microinvasive carcinoma is documented.

Model	Alteration	Driver and/or Add. Genetic Alteration	Phenotype	Reference
*Timp3*	Loss of expression	Homozygous loss of *Pten* (conditional *Pten* allele)	HGPIN with microinvasion	[51]
*mpAkt*	Gain of expression	Myc gain of expression under control of PB driver; loss of Pten function	mPIN followed by microinvasive carcinoma, disruption of basement membrane integrity, stromal remodeling, and lymphocyte infiltration	[52]
*Nkx3.1*	Loss of expression	Myc gain of expression under control of CMV enhancer and β-actin promoter	HGPIN with microinvasion	[53]
*Pten*	Loss of expression	Myc gain of expression under control of CMV enhancer and β-actin promoter	Microinvasive cancer with disruption of smooth muscle actin	[54]

**Table 5:** PCa models in which adenocarcinoma is documented.

Model	Alteration	Driver and/or Add. Genetic Alteration	Phenotype	Reference
*Nkx3.1*	Loss of expression	Hemizygous loss of *Pten* (germline heterozygous *Pten* allele)	HGPIN with invasive adenocarcinoma	[55,56]
*p27^Kip1^*	Loss of expression	Hemizygous loss of *Pten* (germline heterozygous *Pten* allele)	HGPIN with invasive adenocarcinoma	[57,58]
*Aft3*	Loss of expression	Homozygous loss of *Pten* (conditional *Pten* allele)	HGPIN with invasive adenocarcinoma	[59]
*Apc^flox^*	Loss of expression	PB^Cre4^ driver	HGPIN followed by local adenocarcinoma	[60]
*Bmi1*	Gain of expression	Hemizygous loss of *Pten* (germline heterozygous *Pten* allele)	Locally invasive and highly vascularized adenocarcinoma, with frequent bladder outlet obstruction	[61]
*Tsc2*	Loss of expression	Hemizygous loss of *Pten* (germline heterozygous *Pten* allele)	Invasive adenocarcinoma; enhanced lymphoid proliferation; development of skin cancer	[62]
*Phlpp1*	Loss of expression	Hemizygous loss of *Pten* (germline heterozygous *Pten* allele)	Invasive adenocarcinoma at full penetrance with onset of 8 months	[63]
*Chk1*	Loss of expression	Hemizygous loss of *Pten* (germline heterozygous *Pten* allele)	Progression of HGPIN into invasive adenocarcinoma	[64]
*PK* *C* *ε*	Gain of expression	Hemizygous loss of *Pten* (germline heterozygous *Pten* allele)	Invasive adenocarcinoma, preferentially in ventral prostate	[65]
*Gata3*	Loss of expression	Homozygous loss of *Pten* (conditional *Pten* allele)	Acceleration of invasive adenocarcinoma	[66]
*Erg*	Gain of expression	Homozygous loss of *Pten* (conditional *Pten* allele)	Foci of invasive adenocarcinoma with varying levels of *Erg* expression	[67,68]
*Etv1*	Gain of expression	Homozygous loss of *Pten* (conditional *Pten* allele)	Invasive adenocarcinoma with homogenous *Etv1* expression	[69]
*Junb*	Loss of expression	Homozygous loss of *Pten* (conditional *Pten* allele)	Invasive adenocarcinoma in anterior prostate, with strong histological similarity to human PCa	[70]
*SPOP*- *F133V*	Gain of expression	Homozygous loss of *Pten* (conditional *Pten* allele)	Invasive, poorly differentiated, and highly proliferative adenocarcinoma	[71]
*PSGR*	Gain of expression	Homozygous loss of *Pten* (conditional *Pten* allele)	Invasive adenocarcinoma featuring *Akt* activation and extensive inflammatory cell infiltration	[72]
*Zbtb7a*	Loss of expression	Homozygous loss of *Pten* (conditional *Pten* allele)	Highly penetrant invasive adenocarcinoma at 11 weeks	[73]
*Hepsin*	Gain of expression	*Myc* gain of expression under control of PB driver	Invasive adenocarcinoma lacking glandular prostate differentiation and clear basement membrane contour	[74]
*MMP7*	Gain of expression	Loss of Pten function	Invasive adenocarcinoma through induction of epithelial-to-mesenchymal transition (EMT)	[75]
*Pten^adcre+^* ^(1)^	Loss of expression	Cre-expressing adenovirus via intraductal injection into anterior- posterior prostate	Invasive adenocarcinoma with onset of 8–16 weeks	[76]
*Kindlin-3*	Loss of expression	Xenograft	Subcutaneous prostate cancer tumor growth	[77]

^(1)^*Pten^adcre+^*: indicates model with Cre-expressing adenovirus used to disrupt *Pten*.

**Table 7:** PCa models in which metastasis is documented.

Model	Alteration	Driver and/or Add. Genetic Alteration	Phenotype	Reference
*Pten^flox/flox^* (exon 5)	Loss of expression	PB^Cre4^ driver	Invasive adenocarcinoma with metastasis to lungs, rarely to lymph nodes	[91]
*Nr2f2* *(COUP-TFII)*	Gain of expression	Homozygous loss of *Pten* (conditional *Pten* allele)	Invasive adenocarcinoma with metastasis to lymph nodes	[92]
*NCoA2*	Gain of expression	Homozygous loss of *Pten* (conditional *Pten* allele)	Invasive adenocarcinoma with metastasis to lymph nodes, lungs	[93]
*NSD2* *(Whsc-1)*	Gain of expression	Homozygous loss of *Pten* (conditional *Pten* allele)	Invasive adenocarcinoma with metastasis to lymph nodes, lungs, bone	[94]
*Trp53*	Loss of expression	Homozygous loss of *Pten* (conditional *Pten* allele)	Invasive adenocarcinoma with metastasis to lymph nodes, spleen, liver, organs near GU tract excluding bladder	[95,96,97]
*Rb*	Loss of expression	Homozygous loss of *Pten* (conditional *Pten* allele)	Invasive adenocarcinoma with metastasis to lymph nodes, lungs, liver that resembles NEPC	[98]
*Jnk1/2*	Loss of expression	Homozygous loss of *Pten* (conditional *Pten* allele)	Invasive adenocarcinoma with metastasis to lymph nodes	[99]
*Stat3 and* *IL-6*	Loss of expression	Homozygous loss of *Pten* (conditional *Pten* allele)	Poorly differentiated cancer with metastasis to liver, lungs	[100]
*NICD*	Gain of expression	Homozygous loss of *Pten* (conditional *Pten* allele)	Invasive adenocarcinoma with metastasis to liver, lungs	[101]
*Smad4*	Loss of expression	Homozygous loss of *Pten* (conditional *Pten* allele)	Invasive adenocarcinoma with metastasis	[102]
*Smad4/p53*	Loss of expression	Homozygous loss of *Pten* (conditional *Pten* allele)	Invasive adenocarcinoma with metastasis to bone	[103]
*HoxB13/Myc*	Gain of expression	Homozygous loss of *Pten* (conditional *Pten* allele)	Invasive adenocarcinoma with metastasis to lymph nodes, liver, lungs	[104]
*Braf ^V600E (^* ^1)^	Gain of expression	Homozygous loss of *Pten* (conditional *Pten* allele)	Invasive adenocarcinoma with metastasis to lymph nodes, bone marrow, lungs	[105]
*p53^flox^Rb^flox^*	Loss of expression	Homozygous loss of *Pten * (conditional *Pten* allele) PB^Cre4^ driver ^(2)^	Metastatic carcinoma, with distant metastases	[106]
* NPK^EYFP^	**N**kx3.1 loss of expression **P**ten loss of expression **K**ras gain of expression	Nkx3.1^CreERT2/+ (3)^ Pten^flox/flox^ Kras^LSL-G12D/+ (4)^	Invasive adenocarcinoma with metastasis to bone	[107]
*SIRT-6*	Gain of expression	Luciferase expressing PC3M cells in an orthotopic xenograft mouse model	Metastasis to liver; upregulation of N-cadherin and vimentin, downregulation of E-cadherin in vitro	[108]
*RIPK2*	Gain of expression	Injection of RIPK2-KO 22Rv1 cells into male SCID/Beige mice	Invasive adenocarcinoma with metastasis to bone	[109]

^(1)^*Braf ^V600E^*: transgene carrying Braf V600E mutation common in melanoma but used to activate the RAS-MAP kinase pathway; ^(2)^*PB^Cre4^*: probasin promoter; ^(3)^*Nkx3.1-CreER^T2^*: Cre/ERT2 fusion gene (Cre recombinase fused to a human estrogen receptor ligand binding domain) linked to Nkx3.1 gene to drive expression upon tamoxifen induction; ^(4)^*Kras^LSL-G12D/+^*: mice expressing Kras mutant G12D behind the Lox-Stop-Lox (LSL) sequence. Cre recombination deletes the LSL cassette and allows the expression of the mutant KRAS oncogenic protein. * *NPK^EYFP^*: EYFP indicates labeling with enhanced yellow fluorescence protein in the NPK mouse.
